# Effects of Anti-CD20 Antibody Therapy on Immune Cell Dynamics in Relapsing-Remitting Multiple Sclerosis

**DOI:** 10.3390/cells14070552

**Published:** 2025-04-06

**Authors:** Alice G. Willison, Ramona Hagler, Margit Weise, Saskia Elben, Niklas Huntemann, Lars Masanneck, Steffen Pfeuffer, Stefanie Lichtenberg, Kristin S. Golombeck, Lara-Maria Preuth, Leoni Rolfes, Menekse Öztürk, Tobias Ruck, Nico Melzer, Melanie Korsen, Stephen L. Hauser, Hans-Peter Hartung, Philipp A. Lang, Marc Pawlitzki, Saskia Räuber, Sven G. Meuth

**Affiliations:** 1 Department of Neurology, Medical Faculty and University Hospital, Heinrich Heine University Düsseldorf, 40225 Düsseldorf, Germany; 2 Department of Neurology, University Hospital Giessen and Marburg, Justus-Liebig-University Giessen, 35392 Giessen, Germany; 3 Core Facility Flow Cytometry, Medical Faculty, Heinrich Heine University Düsseldorf, 40225 Düsseldorf, Germany; 4 Department of Neurology with Heimer Institute for Muscle Research, University Hospital Bergmannsheil, 44789 Bochum, Germany; 5 UCSF Weill Institute for Neurosciences, Department of Neurology, University of California, San Francisco, CA 94107, USA; 6 Department of Neurology, Medical University of Vienna, 1090 Vienna, Austria; 7 Brain and Mind Center, University of Sydney, Sydney, NSW 2050, Australia; 8 Department of Neurology, Palacky University, 771 46 Olomouc, Czech Republic; 9 Department of Molecular Medicine II, Medical Faculty, Heinrich Heine University, 40225 Düsseldorf, Germany

**Keywords:** multiple sclerosis, autoimmunity, ocrelizumab, ofatumumab, immune reconstitution

## Abstract

Introduction: The efficacy of anti-CD20 antibodies has significantly contributed to advancing our understanding of disease pathogenesis and improved treatment outcomes in relapsing-remitting multiple sclerosis (RRMS). A comprehensive analysis of the peripheral immune cell profile, combined with prospective clinical characterization, of RRMS patients treated with ocrelizumab (OCR) or ofatumumab (OFA) was performed to further understand immune reconstitution following B-cell depletion. Methods: REBELLION-MS is a longitudinal analysis of RRMS patients treated with either OCR (n = 34) or OFA (n = 25). Analysis of B, T, natural killer (NK) and natural killer T (NKT) cells at baseline, month 1, and 12 was performed by multidimensional flow cytometry. Data were analyzed by conventional gating and unsupervised computational approaches. In parallel, different clinical parameters were longitudinally assessed. Twenty treatment-naïve age/sex-matched RRMS patients were included as the control cohort. Results: B-cell depletion by OCR and OFA resulted in significant reductions in CD20^+^ T and B cells as well as B-cell subsets, alongside an expansion of CD5^+^CD19^+^CD20^−^ B cells, while also elevating exhaustion markers (CTLA-4, PD-1, TIGIT, TIM-3) across T, B, NK, and NKT cells. Additionally, regulatory T-cell (T_REG_) numbers increased, especially in OCR-treated patients, and reductions in double-negative (CD3^+^CD4^−^CD8^−^) T cells (DN T cells) were observed, with these DN T cells having higher CD20 expression compared to CD4 or CD8 positive T cells. These immune profile changes correlated with clinical parameters, suggesting pathophysiological relevance in RRMS. Conclusions: Our interim data add weight to the argumentation that the exhaustion/activation markers, notably TIGIT, may be relevant to the pathogenesis of MS. In addition, we identify a potentially interesting increase in the expression of CD5+ on B cells. Finally, we identified a population of double-negative T cells (KLRG1+HLADR+, in particular) that is associated with MS activity and decreased with CD20 depletion.

## 1. Introduction

Multiple sclerosis (MS) is an immune-mediated inflammatory disorder of the central nervous system with a broad spectrum of severity and the potential to cause early-onset long-term disability in a cohort of predominantly young patients. For many years, MS was considered a primarily T-cell-mediated disease. However, the rise in the use of B-cell-depleting therapies for patients with more active disease, specifically monoclonal antibodies against CD20 like ocrelizumab (OCR) and ofatumumab (OFA), has significantly advanced our understanding of the complexity of RRMS pathogenesis and improved treatment outcomes [1]. Both therapies target the CD20 transmembrane protein expressed on the surface of B cells and have demonstrated high efficacy in clinical trials, decreasing disease activity and slowing disease progression [2,3]. Both OCR and OFA are IgG anti-CD20 monoclonal antibodies, with two light and two heavy chains, and both are type I antibodies, meaning they can crosslink two CD20 tetramers and translocate CD20 into lipid rafts, triggering antibody-dependent cellular phagocytosis (ADCC) and complement-dependent cytotoxicity (CDC) against CD20-expressing B cells [4]. OCR primarily induces ADCC, while OFA has a stronger CDC effect, which remains potent even with low CD20 expression [4]. It remains unclear exactly how CD20 depletion and subsequent immune system reconstitution contributes to amelioration of disease in RRMS. Additionally, understanding how long these changes last after treatment with OCR or OFA may aid in refining treatment approaches. This interim analysis from a non-invasive, prospective, observational study (Long-term reconstitution following B-cell depletion in multiple sclerosis (REBELLION-MS; this study is registered at ClinicalTrials.gov under the identifier NCT06586177)) reports peripheral immune reconstitution in the first 12 months of treatment following OCR and OFA therapy and how these changes are associated with the clinical disease course.

## 2. Materials and Methods

### 2.1. Study Population

**Figure 1:**
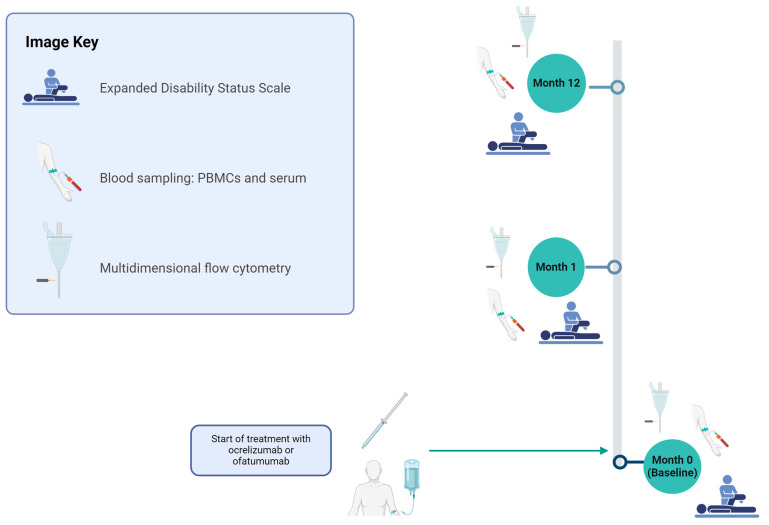
Study design for evaluating OCR and OFA therapies in RRMS patients. The cohort was assessed at months 0, 1, and 12 for the following parameters: basic disease characteristics (only month 0), EDSS, relapse documentation, and immunological analyses using PBMCs. Figure 1 was created in BioRender (https://biorender.com/, last accessed on 27 March 2025) Willison, A. (2024) BioRender.com/n12e680 [6]. EDSS—expanded disability status scale; OCR—ocrelizumab; OFA—ofatumumab; PBMCs—peripheral blood mononuclear cells.

Patients with a diagnosis of RRMS according to the 2017 McDonald criteria [5] were prospectively included in this study and divided into three groups: ocrelizumab, ofatumumab, and treatment-naïve relapsing-remitting multiple sclerosis (tnRRMS). The exclusion criteria were as follows: prior treatment with B cell-modulating therapies; any previous use of alemtuzumab, cyclophosphamide, mitoxantrone, azathioprine, mycophenolate mofetil, cyclosporine, methotrexate, total body irradiation, or bone marrow transplantation; impaired decision-making or consent capacity; ongoing immunosuppressive treatment for conditions other than RRMS; confirmed human immunodeficiency virus (HIV) or active/chronic hepatitis B/C infection. The decision to start OCR or OFA treatment was made independently of study inclusion. According to the Summary of Product Characteristics (SmPC), the dosage of subcutaneous ofatumumab is as follows: 20 mg at weeks 0, 1, and 2 followed by subsequent doses of 20 mg every 4 weeks, starting at week 4 and the dosage of intravenous ocrelizumab as follows: 300 mg with a repeat dose of 300 mg 2 weeks later, then 600 mg every 6 months. A simplified study design is depicted in Figure 1. At month 1 of treatment (m1), 16 patients from the OCR group and 22 from the OFA group were assessed. At the time of data analysis, 24 patients in the OCR group and 17 patients in the OFA group had reached the 12-month (m12) follow-up point for assessment and were included in this interim analysis.

### 2.2. Isolation of PBMCs and mFC Analysis (In-Depth mFC Cohort)

Blood samples for the study were collected concurrently with routine clinical blood draws. Peripheral blood mononuclear cells (PBMCs) were isolated from whole blood by Ficoll gradient with SepMate isolation tubes (StemCell Technologies, Vancouver, Canada) and were cryopreserved in liquid nitrogen. The samples were prepared for multidimensional flow cytometry (mFC) per standard protocol [7] using the fluorochrome-conjugated antibodies detailed in Supplementary Table S1. For intracellular staining (FoxP3), the Foxp3/Transcription Factor Staining Buffer Set (eBioscience, San Diego, CA, USA) was used following cell surface marker staining according to the manufacturer’s instructions.

A CytoFLEX-S (Beckman Coulter) was used to acquire data. Manual gating was performed with the software ‘Kaluza Flow Cytometry Analysis’ version 2.1 (Supplementary Table S1, Supplementary Figures S1–S5). The percentage of all living cells was calculated for every cell population and was compared among groups. Furthermore, unsupervised analysis was performed using the platform OMIQ from Dotmatics (www.omiq.ai, www.dotmatics.com, last accessed on 27 March 2025) [8]. For this, compensated, pre-gated event data (CD19^+^ B cells or CD3^+^ lymphocytes) were exported as .csv files with the software ‘Kaluza Flow Cytometry Analysis’. The gating strategies employed for cell identification were as follows: CD19+ B cells were identified using the sequence: singlets → living cells → lineage-negative (CD3-/CD14-/CD56-) → CD19+. For CD3+ T cells, the gating sequence was singlets → living cells → CD3+ lymphocytes. To maximize sensitivity and ensure comprehensive data acquisition, the CytoFLEX-S flow cytometer was manually monitored during data collection, and the flow rate was adjusted to a low setting to ensure optimal detection and minimize cell loss, allowing, insofar as possible, the entire prepared sample volume to pass through the laser for analysis. The event data and the corresponding metadata were uploaded to the platform OMIQ. Optimized t-distributed Stochastic Neighbor Embedding (Opt-SNE) plots using the mFC data from all three groups were created using the default parameters (max iterations = 1000, opt-SNE end = 5000, perplexity = 30, theta = 0.5, components = 2, random seed = 1759, verbosity = 25). The algorithm FlowSOM (xdim = 12, ydim = 12, rlen = 10, distance metric euclidean) was used for cluster identification. A clustered heatmap of concatenated files was created to visualize the median marker expression of each cluster.

### 2.3. Data Analysis

RStudio (2023.06.1) was used for data analysis and visualization. *p*-values were calculated using analysis of variance (ANOVA) with post-hoc Tukey Honestly Significant Difference (HSD), if normality could be assumed based on the Shapiro–Wilk test, otherwise, the Kruskal–Wallis test was used with the Dunn post hoc test (p-adjustment method: Benjamini–Hochberg). A *p*-value of < 0.05 was considered statistically significant. Violin plots were created with the R package ‘ggplot2’ (v3.4.4). R packages ‘umap’ (v0.2.10.0) and ‘ggplot2’ (v3.4.4) were used to create UMAPs (uniform manifold approximation and projection for dimension reduction). To perform correlation analyses, Spearman correlation coefficients were calculated as normality of data could not be assumed. Correlation plots were created with ‘ggplot2’ (v3.4.4). Correlation was performed between the CD5 mean fluorescent intensity (MFI) of B cells, TIM-3, TIGIT, PD-1, and CTLA-4 MFIs of B cells, T cells, NK cells, and NKT cells, as well as percentages of regulatory T cells (T_REG_), B cell cluster 16, T cell cluster 8, 11, 13, 15, and different clinical parameters (the Expanded Disability Status Scale (EDSS)), time since disease manifestation, time since last relapse, the annual relapse rate prior to sampling, and the total number of previous relapses). The graphical data were processed and refined for presentation using Inkscape (Harrington, B. et al., 2004–2005), a vector graphics software, to create the figures (Inkscape, http://www.inkscape.org/, last accessed on 27 March 2025) [9].

## 3. Results

### 3.1. Study Population

34 OCR-treated patients, 25 OFA-treated patients, and 20 tnRRMS patients were included in the analysis. The median age of participants was 36 years (range 19–55) in the OCR group, 37 years (range 22–54) in the OFA group, and 37 years (range 19–57) in the tnRRMS group. The percentage of female patients was 71% in the OCR group, 80% in the OFA group, and 90% in the treatment-naïve group. The median disease duration was 3.42 years in both the OCR (range 0.17–22.08) and OFA (range 0.08–25.33) groups, while it was 0.63 years (range 0.00–19.67) in the treatment-naïve group. Baseline EDSS scores had a median of 2.00 (range 0.0–6.5) in the OCR group, 2.00 (range 0.0–3.5) in the OFA group, and 1.25 (range 0.0–5.0) in the treatment-naïve group. The median annualized relapse rate (ARR) at baseline (the number of relapses in the year prior to treatment) was 1 (range 0–2) for OCR, 1 (range 0–3) for OFA, and 1 (range 0–3) for the treatment-naïve group. The median number of previous disease-modifying therapies (DMTs) was 1 (range 0–4) in the OCR group, 1 (range 0–5) in the OFA group, and 0 (range 0) in the tnRRMS group (Table 1). The last previous DMTs were glatiramer acetate in six patients (four OFA, two OCR), dimethyl fumarate in four patients (one OFA, three OCR), interferon in four patients (one OFA, three OCR), cladribine in one OFA patient, teriflunomide in six patients (three OFA, three OCR), natalizumab in eight patients (three OFA, five OCR), and fingolimod in five patients (two OFA, three OCR). A total of 25 patients (10 OFA, 15 OCR) were treatment-naïve prior to OFA or OCR initiation (Supplementary Table S2).

### 3.2. Effective Depletion of B-Cell Subsets and CD20^+^ T Cells Occurs Following Treatment with Anti-CD20-Antibodies

**Figure 2:**
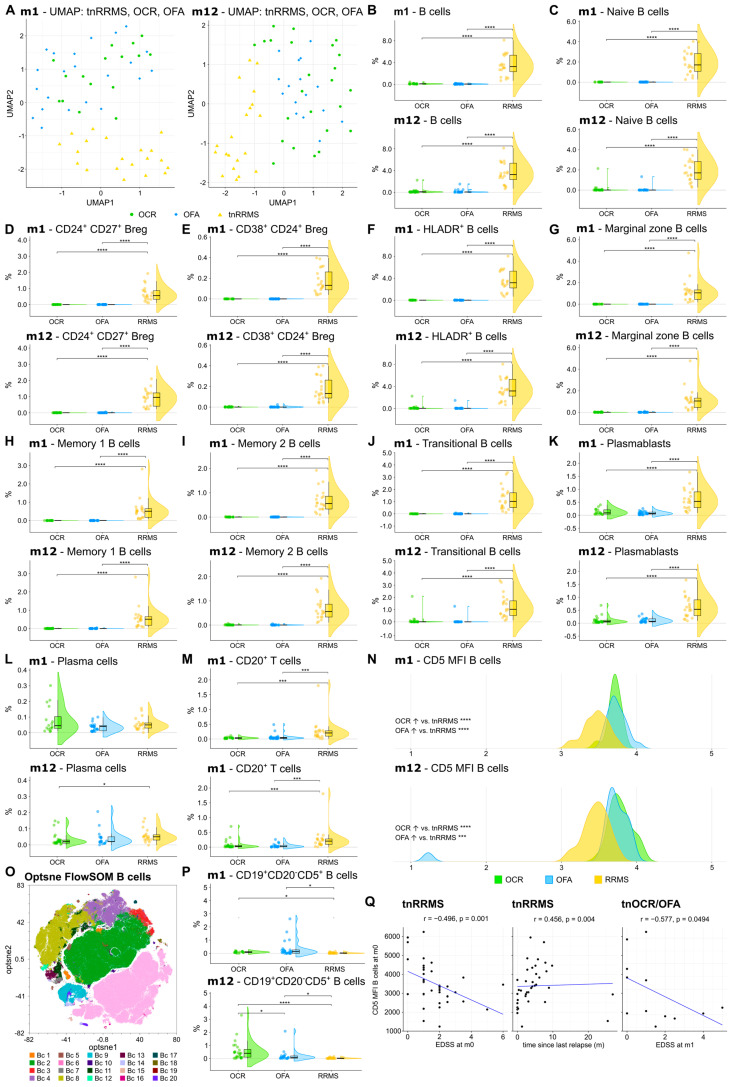
Reduction in CD20^+^ B and T cells, while CD5 expression was increased on B cells of OCR- and OFA-treated compared to treatment-naïve RRMS patients. (**A**) UMAP analysis including the PB immune cell subsets assessed by mFC. Each colored symbol presents an individual patient. (**B**–**M**) Violin plots with overlaying boxplots illustrating CD20⁺ B cells, CD20+ T cells, and various B cell subsets (% of living cells) in the PB of OCR-treated, OFA-treated, and treatment-naïve RRMS patients. Boxes show the median and the 25th as well as the 75th percentiles. The whiskers extend from the hinge to the largest and smallest values, respectively, but no further than 1.5 * IQR from the hinge. *p*-values were calculated using analysis of variance (ANOVA) with post hoc Tukey Honestly Significant Difference (HSD), if normality could be assumed based on the Shapiro–Wilk test, otherwise the Kruskal–Wallis test was used with the Dunn post hoc test (p-adjustment method: Benjamini–Hochberg). (**N**) Ridge plots illustrating the MFI of CD5 on B cells of OCR- and OFA-treated compared to treatment-naïve RRMS patients. *p*-values were calculated using the Kruskal–Wallis test with the Dunn post hoc test (p-adjustment method: Benjamini–Hochberg) as normality could not be assumed based on the Shapiro–Wilk test. (**O**) Opt-SNE plots with B-cell clusters identified by FlowSOM. Opt-SNE plot including mFC data from OCR-treated, OFA-treated, and treatment-naïve RRMS patients were created with the platform OMIQ from Dotmatics (www.omiq.ai, www.dotmatics.com, last accessed on 27 March 2025) [8] using the default parameters (max iterations = 1000, opt-SNE end = 5000, perplexity = 30, theta = 0.5, components = 2, random seed = 1759, verbosity = 25). The algorithm FlowSOM (xdim = 12, ydim = 12, rlen = 10, distance metric euclidean) was used for cluster identification. (**P**) Violin plots with overlaying box plots depicting CD19^+^CD20^−^CD5^+^ B cells (% of living cells) identified by FlowSOM. Boxes show the median and the 25th as well as the 75th percentiles. The whiskers extend from the hinge to the largest and smallest values, respectively, but no further than 1.5 * IQR from the hinge. *p*-values were calculated using the Kruskal–Wallis test with the Dunn post hoc test (p-adjustment method: Benjamini–Hochberg) as normality could not be assumed based on the Shapiro–Wilk test. (**Q**) Correlation analysis between the CD5 MFI of B cells and EDSS as well as time since last relapse. Spearman correlation coefficients were calculated as normality of data could not be assumed. Bc—B cell; Breg—regulatory B cells; EDSS—expanded disability status scale; m—month(s); MFI—mean fluorescent intensity; OCR—ocrelizumab; OFA—ofatumumab; PB—peripheral blood; tnRRMS—treatment-naïve relapsing-remitting multiple sclerosis; tnOCR/OFA—OCR- and OFA-treated RRMS patients who were treatment-naïve at m0, UMAP: Uniform Manifold Approximation and Projection for Dimension Reduction. Statistical significance: *p* ≤ 0.05 (*), *p* ≤ 0.001 (***), *p* ≤ 0.0001 (****).

We first explored the effect of OCR and OFA on different B-cell subsets and CD20^+^ T cells, compared to the tnRRMS patients. UMAP analysis demonstrated longitudinal clustering of OFA- and OCR-treated patients, indicating similarities in the peripheral immune cell profile, distinct from tnRRMS (Figure 2A). Compared to the tnRRMS patients at m1 and m12, a significant reduction in CD20^+^ B cells and all analyzed B-cell subsets (naïve B cells, CD24^+^CD27^+^ regulatory B cells (Bregs), CD38^+^CD24^+^ Bregs, HLADR^+^ B cells, marginal zone B cells, memory B cells, transitional B cells), as well as in plasmablasts occurred (Figure 2B–K). Plasma cell populations were expectedly not dramatically affected by B-cell depletion (Figure 2L). In addition, the percentage of CD3^+^CD20^+^ T cells significantly decreased at m1 and m12 (Figure 2M).

### 3.3. CD5^+^CD19^+^ B Cell Population Increases Following Anti-CD20 Antibody Treatment

A significant increase in CD5 expression was observed on the remaining B cells at m1 and m12 in OCR- and OFA-treated RRMS patients compared to tnRRMS (Figure 2N). Unsupervised clustering was performed on the B-cell population (Figure 2O), which identified the expansion of a CD19^+^CD20^−^CD5^+^ B-cell population in OCR- and OFA-treated patients at m1 and m12 compared to tnRRMS patients (Figure 2P). A significantly larger population expansion was observed in the OCR- compared to OFA-treated and tnRRMS patients at m12, which was not observed at m1. We found a negative correlation between CD5 expression on B cells and EDSS at m0 (correlation coefficient r = −0.496, *p* = 0.001) and a positive correlation with the time since the last relapse (r = 0.456; *p* = 0.004) in tnRRMS patients (Figure 2Q). In addition, we observed a negative correlation between CD5 expression on B cells in treatment-naïve OCR and OFA patients at m0 and EDSS at m1 (r = −0.577, *p* = 0.0494) (Figure 2Q).

### 3.4. Increased Expression of Exhaustion/Activation Marker on Immune Cells Following Anti-CD20 Antibody Treatment

**Figure 3:**
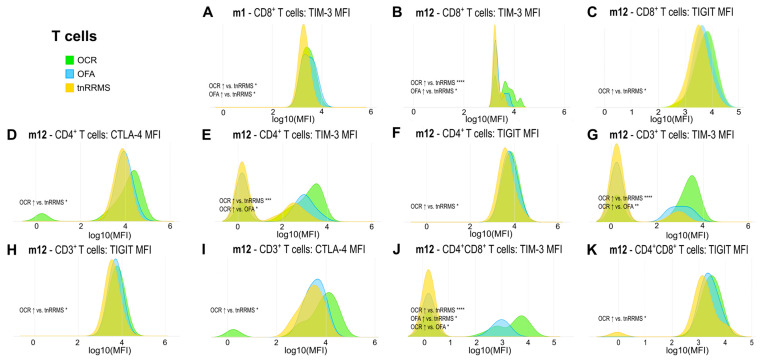
Increased expression of exhaustion/activation markers on T cells of OCR- and OFA-treated RRMS patients. Ridge plots illustrating the MFIs of different exhaustion/activation markers on T cells (**A**–**K**) of OCR- and OFA-treated compared to treatment-naïve RRMS patients. *p*-values were calculated using analysis of variance (ANOVA) with post-hoc Tukey Honestly Significant Difference (HSD), if normality could be assumed based on the Shapiro–Wilk test, otherwise the Kruskal–Wallis test was used with the Dunn post hoc test (p-adjustment method: Benjamini–Hochberg). m—month(s); MFI—mean fluorescent intensity; OCR—ocrelizumab; OFA—ofatumumab; RRMS—relapsing-remitting multiple sclerosis; tnRRMS—treatment-naïve relapsing-remitting multiple sclerosis. Statistical significance: *p* ≤ 0.05 (*), *p* ≤ 0.01 (**), *p* ≤ 0.001 (***), *p* ≤ 0.0001 (****).

**Figure 4:**
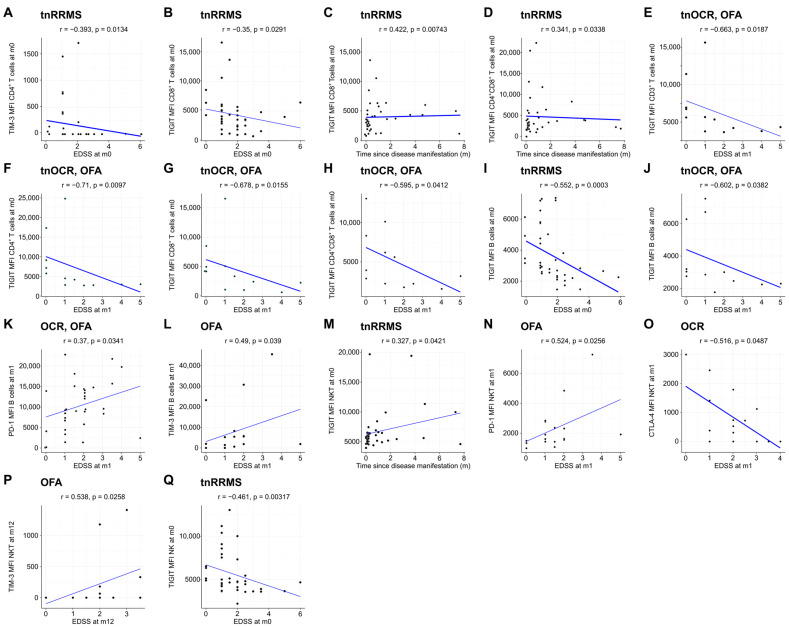
Expression of exhaustion/activation markers on immune cells correlates with clinical parameters of RRMS patients. Correlation analysis between different exhaustion/activation markers on T cells (**A**–**H**), B cells (**I**–**L**), NKT cells (**M**–**P**), and NK cells (**Q**) and EDSS as well as time since disease manifestation. Spearman correlation coefficients were calculated as normality of data could not be assumed. EDSS—expanded disability status scale; m—month(s); MFI—mean fluorescent intensity; NK—natural killer cells; OCR—ocrelizumab; OFA—ofatumumab; RRMS—relapsing-remitting multiple sclerosis; tnOCR/OFA—OCR- and OFA-treated RRMS patients who were treatment- naïve at m0; tnRRMS—treatment-naïve relapsing-remitting multiple sclerosis.

Moreover, we identified an increase in TIM-3, TIGIT, and CTLA-4 expression on T cells in B-cell-depleted patients, particularly in the OCR group, with correlations observed between these markers and clinical parameters in tnRRMS patients. At m1, TIM-3 expression on CD8^+^ T cells was higher in B-cell-depleted patients compared to tnRRMS (Figure 3A). At m12, this increase was more pronounced in the OCR group than OFA but remained significant for both (Figure 3B). TIGIT expression on CD8^+^ T cells also increased in the OCR group compared to tnRRMS at m12 (Figure 3C). On CD4^+^ T cells at m12, significantly higher expression of CTLA-4, TIM-3, and TIGIT in the OCR group compared to tnRRMS was observed, with TIM-3 expression in the OFA group also reaching significantly higher expression at m12 (Figure 3D–F). This pattern was also seen in CD3^+^ T cells (Figure 3G–I). At m12 on CD4^+^CD8^+^ T cells, TIM-3 expression in the OCR group was markedly increased compared to tnRRMS and to a lesser extent when compared to the OFA group; comparing OFA to tnRRMS, the increase in TIM-3 expression was also observed (Figure 3J). TIGIT expression on CD4^+^CD8^+^ T cells increased in the OCR group compared to tnRRMS at m12 (Figure 3K). At baseline in tnRRMS patients, TIM-3 expression on CD4^+^ T cells and TIGIT expression on CD8^+^ T cells correlated negatively with EDSS (r = −0.393, *p* = 0.013 and r = −0.35, *p* = 0.029) (Figure 4A,B). In addition, TIGIT expression on CD8^+^ T cells and on CD4^+^CD8^+^ T cells was positively correlated with the time since disease manifestation (r = 0.422, *p* = 0.007 and r = 0.341, *p* = 0.034) (Figure 4C,D). Furthermore, we observed a negative correlation between TIGIT expression on T cells (CD3^+^, CD4^+^, CD8^+^, CD4^+^CD8^+^) in treatment-naïve OCR und OFA patients at baseline and EDSS at m1 (r = −0.663, *p* = 0.0187; r = −0.71, *p* = 0.0097; r = −0.678, *p* = 0.0155; r = −0.595, *p* = 0.0412) (Figure 4E–H).

**Figure 5:**
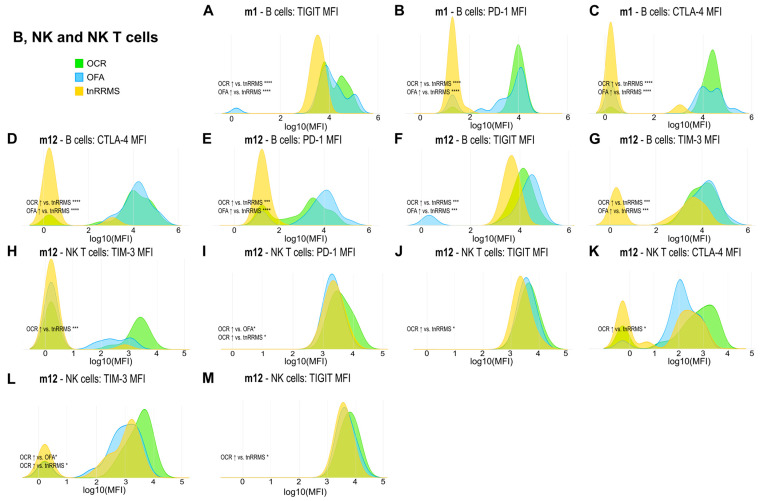
Higher expression of exhaustion/activation markers on B, NK, and NKT cells of OCR- and OFA-treated RRMS patients. Ridge plots illustrating the MFIs of different exhaustion/activation markers on B, NK, and NKT cells (**A**–**M**) of OCR- and OFA-treated compared to treatment-naïve RRMS patients. *p*-values were calculated using analysis of variance (ANOVA) with post hoc Tukey Honestly Significant Difference (HSD), if normality could be assumed based on the Shapiro–Wilk test, otherwise the Kruskal–Wallis test was used with the Dunn post hoc test (p-adjustment method: Benjamini–Hochberg). m—month(s); MFI—mean fluorescent intensity; NK—natural killer cells; OCR—ocrelizumab; OFA—ofatumumab; RRMS—relapsing-remitting multiple sclerosis; tnRRMS—treatment-naïve relapsing-remitting multiple sclerosis. Statistical significance: *p* ≤ 0.05 (*), *p* ≤ 0.001 (***), *p* ≤ 0.0001 (****).

Beyond the increased expression of exhaustion/activation marker on T cells of B-cell-depleted patients, we observed significant changes in their expression levels on B, NK, and NKT cells. At m1, expression of TIGIT, PD-1, and CTLA-4 on B cells was increased in both OCR- and OFA-treated patients compared to tnRRMS (Figure 5A–C). This was also observed at m12, with expression of TIM-3 also significantly increased at this time point (Figure 5D–G). On NKT cells at m12, expression of TIM-3, PD-1, TIGIT, and CTLA-4 was higher in OCR-treated patients compared to tnRRMS (Figure 5H–K), with PD-1 expression also higher than in OFA-treated patients (Figure 5I). Expression of TIM-3 and TIGIT was also higher on NK cells in OCR-treated patients compared to tnRRMS at m12 (Figure 5L,M), with TIM-3 expression higher in OCR- compared to OFA-treated patients (Figure 5L). At baseline in tnRRMS patients, TIGIT expression on B cells negatively correlated with EDSS (r = −0.552, *p* = 0.0003) (Figure 4I). In the treatment naïve OCR- and OFA-treated patients, TIGIT expression on B cells at m0 negatively correlated with EDSS at m1 (r = −0.602, *p* = 0.0382) (Figure 4J). In OCR- and OFA-treated patients, EDSS at m1 positively correlated with PD-1 expression on B cells at m1 (r = 0.37, *p* = 0.0341) (Figure 4K). EDSS at m1 also correlated positively with TIM-3 expression on B cells at m1 in OFA-treated patients (r = 0.49, *p* = 0.039) (Figure 4L). TIGIT expression on NKT cells at baseline positively correlated with time since disease manifestation (r = 0.327, *p* = 0.042) (Figure 4M). In OFA-treated patients, PD-1 expression on NKT cells at m1 positively correlated with EDSS at m1 (r = 0.524, *p* = 0.0256) (Figure 4N). EDSS at m1 negatively correlated with CTLA-4 expression on NKT cells at m1 in OCR-treated patients (r = −0.516, *p* = 0.0487) (Figure 4O). In OFA-treated patients, TIM-3 expression at m12 positively correlated with EDSS at m12 (r = 0.538, *p* = 0.0258) (Figure 4P). TIGIT expression on NK cells at baseline negatively correlated with EDSS at baseline (r = −0.461, *p* = 0.003) (Figure 4Q).

### 3.5. An Increase in Regulatory T Cells and Decrease in Double Negative T Cell Subsets in OCR- and OFA-Treated RRMS Patients

**Figure 6:**
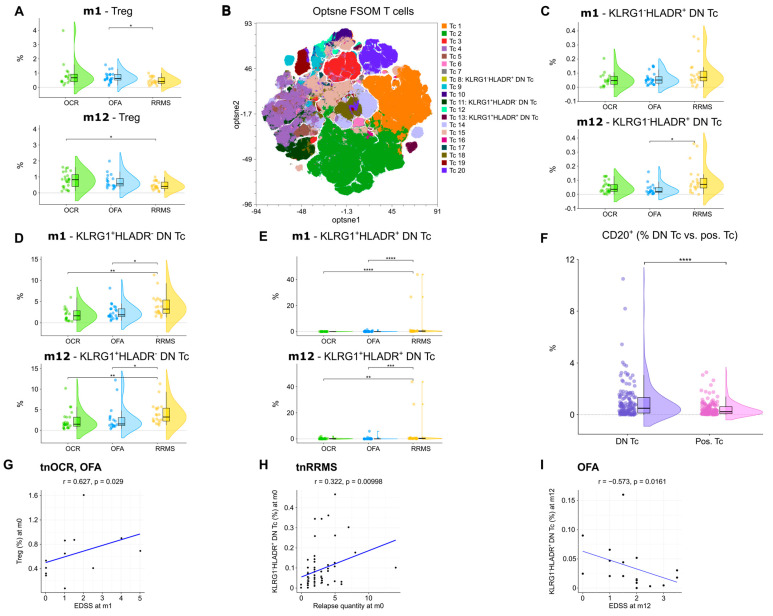
Increase in regulatory T cells and reduction in double negative T cells in OCR- and OFA-treated RRMS patients. (**A**) Violin plots with overlaying boxplots showing T_REG_s (% of living cells) in the PB of OCR-treated, OFA-treated, and treatment-naïve RRMS patients. Boxes show the median and the 25th as well as the 75th percentiles. The whiskers extend from the hinge to the largest and smallest values, respectively, but no further than 1.5 * IQR from the hinge. *p*-values were calculated using the Kruskal–Wallis test with the Dunn post hoc test (p-adjustment method: Benjamini–Hochberg) as normality could not be assumed based on the Shapiro–Wilk test. (**B**) Opt-SNE plots with T cell clusters identified by FlowSOM. Opt-SNE plot including mFC data from OCR-treated, OFA-treated, and treatment-naïve RRMS patients were created with the platform OMIQ from Dotmatics (www.omiq.ai, www.dotmatics.com) using the default parameters (max iterations = 1000, opt-SNE end = 5000, perplexity = 30, theta = 0.5, components = 2, random seed = 1759, verbosity = 25). The algorithm FlowSOM (xdim = 12, ydim = 12, rlen = 10, distance metric euclidean) was used for cluster identification. (**C**–**E**) Violin plots with overlaying box plots depicting KLRG1^−^HLADR^+^ DN T cells (**C**), KLRG1^+^HLADR^−^ DN T cells (**D**), and KLRG1^+^HLADR^+^ DN T cells (**E**). Boxes show the median and the 25th as well as the 75th percentiles. The whiskers extend from the hinge to the largest and smallest values, respectively, but no further than 1.5 * IQR from the hinge. *p*-values were calculated using analysis of variance (ANOVA) with post hoc Tukey Honestly Significant Difference (HSD), if normality could be assumed based on the Shapiro–Wilk test, otherwise the Kruskal–Wallis test was used with the Dunn post hoc test (p-adjustment method: Benjamini–Hochberg). (**F**) Violin plots with overlaying boxplots illustrating the percentage of CD20^+^ T cells among all DN T cells compared to the percentage of CD20^+^ T cells among all CD4^+^ and/or CD8^+^ T cells. Boxes show the median and the 25th as well as the 75th percentiles. The whiskers extend from the hinge to the largest and smallest values, respectively, but no further than 1.5 * IQR from the hinge. *p*-values were calculated with paired Wilcoxon’s rank-sum test. (**G**–**I**) Correlation analysis between T_REG_s and EDSS at m1 (**G**), KLRG1^−^HLADR^+^ DN T cells and relapse quantity at m0 (**H**), as well as KLRG1^−^HLADR^+^ DN T cells and EDSS at m12 (**I**). Spearman correlation coefficients were calculated as normality of data could not be assumed. DN Tc—CD4^−^CD8^−^ T cells; EDSS—expanded disability status scale; m—month(s); OCR—ocrelizumab; OFA—ofatumumab; pos. Tc—CD4^+^ and/or CD8^+^ T cells; PB—peripheral blood; RRMS—relapsing-remitting multiple sclerosis; tnRRMS—treatment-naïve relapsing-remitting multiple sclerosis; tnOCR/OFA—OCR- and OFA-treated RRMS patients who were treatment- naïve at m0; Tc—T cells; Treg—regulatory T cells. Statistical significance: *p* ≤ 0.05 (*), *p* ≤ 0.01 (**), *p* ≤ 0.001 (***), *p* ≤ 0.0001 (****).

An increase in regulatory T cells (T_REG_) at m1 was observed in OFA-treated patients compared to tnRRMS (Figure 6A). At m12, T_REG_ were higher in both OCR- and OFA-treated patients compared to those with tnRRMS; however, significance was only reached in the OCR-treated patients (Figure 6A). Unsupervised clustering was performed on the CD3^+^ T cell population (Figure 6B), allowing for the identification of three subsets of double negative (CD4^−^CD8^−^) T cells (DN T cells), which were reduced in OCR- and OFA-treated RRMS compared to tnRRMS patients. The percentage of KLRG1^−^HLADR^+^ DN T cells decreased in OFA-treated patients compared to tnRRMS at m12 (Figure 6C). The percentage of KLRG1^+^HLADR^−^ DN T cells and KLRG1^+^HLADR^+^ DN T cells in patients treated with B cell depletion decreased significantly compared to tnRRMS patients at both m1 and m12 (Figure 6D,E). Compared to CD4 and/or CD8 positive T cells, significantly more DN T cells expressed CD20 (Figure 6F). Furthermore, we observed a positive correlation between T_REG_ in treatment-naïve OCR and OFA patients at baseline and EDSS at m1 (r = 0.627, *p* = 0.029) (Figure 6G). The percentage of KLRG1^−^HLADR^+^ DN T cells correlated positively with the number of relapses prior to baseline (r = 0.322, *p* = 0.01) (Figure 6H). In addition, we observed a negative correlation between KLRG1^−^HLADR^+^ DN T cells in OFA-treated patients at m12 and EDSS in this group at m12 (r = −0.573, *p* = 0.0161) (Figure 6I).

## 4. Discussion

Previous studies have characterized the repopulation of immune cells following B-cell depletion, with a particular focus on how the T-cell compartment repopulates [10,11,12,13,14]. Expanding on these observations, we used a comprehensive panel of antibodies to characterize immune reconstitution in detail following B-cell depletion. We found that B-cell depletion by OCR and OFA resulted in significant reductions in CD20^+^ T and B cells. alongside an expansion of CD5^+^CD19^+^CD20^−^ B cells, particularly in OCR-treated patients at 12 months. Exhaustion/activation markers (CTLA-4, PD-1, TIGIT, TIM-3) across T, B, NK, and NKT cells were increased in anti-CD20 treated individuals. Expression of TIGIT generally demonstrated correlations with disease parameters that indicate a positive effect on disease course. Clinical correlations of PD-1, TIM-3, and CTLA-4 expression did not demonstrate a clear effect on disease course. Additionally, T_REG_s increased, particularly in OCR-treated patients, and a reduction in potentially disease-relevant double-negative (CD4^−^CD8^−^) T-cell subsets were observed.

### 4.1. The Relevance of CD5^+^ B Cells in MS

The CD5-expressing B-cell subsets are considered a complex, heterogeneous population of human B cells involved in ‘natural’ or polyreactive innate immunity, B-cell stimulation and autoreactive antibody production [15,16]. CD5^+^ murine B-1 cells have been defined as innate, natural-antibody-producing immature cells with a limited repertoire of V genes and less junctional diversity than bone marrow-derived B-2 cells. Human B1-like CD5^+^ cells have been found in human fetal lymph nodes [17], spleen [18], and umbilical vein cord blood [19], but due to the phenotypical overlap with activated B cells, this natural population of B-1-like cells is difficult to characterize in humans. CD5^+^ B cells have, however, been implicated in the pathogenesis and regulation of MS as detailed below.

In mouse models, a protective role of CD5^+^ B cells has been observed. Yanaba et al. identified a distinct regulatory subset of CD1d(hi)CD5^+^ B cells that modulated T cell-mediated inflammation [20]. Interleukin (IL)-10 production was restricted to this subset and diminished in CD19-deficient mice. Adoptive transfer of this B-cell population to the CD19-deficient and wild-type mice normalized T-cell-mediated inflammation, supporting the regulatory potency of IL-10-producing CD5^+^ B cells [20]. Niino et al. demonstrated that in patients with MS, the percentage of CD5^+^ cells in the memory B cell (CD27^+^) subset is significantly higher during the remitting stage compared to the relapsing stage [21]. In a later publication, Niino et al. observed that CD5 expression on B cells decreases notably in secondary progression MS (SPMS) patients [22].

However, CD5 expression on B cells has also been demonstrated to be significantly higher in patients with active MS compared to those with stable disease, correlating with active lesions and myelin basic protein (MBP) antibodies [23]. Villar et al. investigated the role of oligoclonal immunoglobulin M (IgM) bands in cerebrospinal fluid (CSF) as a prognostic marker in MS and identified CD5^+^ B cells as the subpopulation responsible for secreting IgM antibodies, particularly against non-protein molecules like myelin lipids. They found that patients with these anti-lipid IgM antibodies experienced more aggressive disease [24]. Fluorescence-activated cell sorting (FACS) analysis of CSF and peripheral blood by Correale et al. demonstrated that CD5^+^CD19^+^ B cells were increased intrathecally in MS compared to control and that HLA-DR expression was lower in CD5^+^ B cells than CD5^−^ B cells. The authors therefore viewed the function of CD5^+^ B cells to be intrathecal autoantibody production and not presentation [25].

The increase in CD5 expression on B cells at both m1 and m12 after B-cell depletion that we observed in our analysis suggests a compensatory or adaptive role for this population following B cell-targeted therapies. The more significant expansion observed in OCR-treated patients at m12 could indicate a differential effect of OCR compared to OFA on the B cell compartment. The negative correlation between CD5 expression and EDSS suggests that higher CD5 expression on B cells might be associated with less disability. This could indicate a protective or regulatory role for CD5^+^ B cells. The positive correlation between CD5 expression and the time since the last relapse suggests that CD5 positivity on B cells might contribute to disease quiescence. These findings resonate with previous studies that have highlighted the complex role of CD5^+^ B cells in MS. The expansion of this cell population following B cell depletion suggests that they could be involved in re-establishing immune balance after such therapies, and our population may represent B10 cells or B regulatory cells [26].

### 4.2. CTLA-4, TIGIT, TIM-3, and PD-1 Expression in MS

CTLA-4, TIGIT, TIM-3, and PD-1 are markers of immune cell exhaustion and activation [27,28,29,30,31]. We found significant increases in CTLA-4, TIGIT, TIM-3, and PD-1 across CD4^+^, CD8^+^ T cells, B cells, NK cells, and NKT cells following B cell-depleting therapies compared to tnRRMS patients.

In MS patients, compared to healthy controls, expression of CTLA-4, PD-1, and TIM-3 has been found to be downregulated [32]. Asashima et al. explored the role of B-cell function in RRMS and found that impaired TIGIT expression may drive disease pathogenesis [33]. Trabattoni et al. investigated costimulatory molecule expression and cytokine production in patients with RRMS, finding that PD-1-expressing T cells and PD-L1-expressing B cells and monocytes were significantly elevated in stable MS compared to acute MS. This upregulation of the PD-1/PD-L1 pathway during stable phases of MS was linked to enhanced IL-10 production and reduced proliferation of MBP-specific CD4^+^ and CD8^+^ T cells [34]. Yang et al. found that TIM-3 is dysregulated in untreated MS patients, leading to impaired TIM-3-mediated immunoregulation. Blocking TIM-3 in healthy controls increased IFN-γ secretion, but this effect was absent in untreated MS patients, indicating a defect that was reversed by treatment with glatiramer acetate or IFN-β, implying that patients with MS have impaired TIM-3 expression. The restoration of TIM-3 expression and function in treated patients suggests that these therapies may partially work by restoring TIM-3-mediated regulation of T-cell function [35]. Koguchi et al. generated 104 T cell clones from the CSF of six patients with MS. They found that MS CSF clones, despite secreting higher levels of IFN-γ, paradoxically exhibited lower TIM-3 and T-bet expression compared to controls. IL-12 polarization further increased IFN-γ secretion while reducing TIM-3 levels in MS clones, indicating dysregulated TIM-3 expression. Reduced TIM-3 expression was linked to resistance to tolerance and enhanced T-cell proliferation and IFN-γ secretion, suggesting that impaired upregulation of TIM-3 in inflammatory sites may be an intrinsic defect contributing to MS pathogenesis [36]. The literature in this field of research raises interesting questions, and further characterization of immune checkpoint proteins in MS is ongoing–and necessary.

Relapse or first presentation of MS is a rare but serious complication following immune checkpoint inhibitor (ICI) treatment. In an analysis of the FDA Adverse Event Reporting System (FAERS) database and additional case reports, 14 cases of MS were identified in patients who had received an ICI for metastatic cancer [37]. In all cases, ICI therapy was discontinued upon clear clinical diagnosis of MS or MS relapse. One patient was re-treated with the CTLA-4-blocking antibody ipilimumab after a mild MS relapse due to the favorable cancer response and experienced significantly worsened MS symptoms, leading to discontinuation of the retreatment. In two cases, the MS relapse was severe, marked by rapid progression and poor response to MS therapy [38]. Gerdes et al. presented a case study of a patient who developed MS during treatment with ipilimumab. After two courses of ipilimumab, the patient experienced clinical episodes of MS, with one episode showing a marked increase in MRI activity. A brain biopsy confirmed active, T cell-mediated MS [39]. However, the ACCLAIM study did not demonstrate an effect of abatacept (CTLA-4-Ig) on the number of new gadolinium-enhancing MRI lesions, or clinical measures of disease activity in RRMS [40].

Our findings contribute to the growing body of evidence that immune checkpoint markers, particularly CTLA-4, TIGIT, TIM-3, and PD-1, may be significant in the immunopathology of MS. Clinical correlations demonstrated a possibly protective effect of TIGIT expression. A clear effect of CTLA-4, TIM-3, and PD-1 on disease course was not observed in this study. Impaired expression of these markers, notably TIGIT, may impact MS severity.

### 4.3. CD20^+^ T Cells, Regulatory T Cells, and Double Negative T-Cell Subsets

CD20^+^ T cells have been implicated in the pathogenesis of MS through cytokine production, reduction under disease-modifying therapies, association with white matter injury, and presence intrathecally [41,42,43]. We demonstrate that CD20+ T cells are depleted by both OFA and OCR, which may contribute to their therapeutic efficacy. T_REG_s regulate the number and function of autoreactive T cells, with clear relevance to the pathophysiology of MS. In line with our findings, increased T_REG_ frequencies following CD20^+^ depletion have been reported, indicating a shift towards an anti-inflammatory state [44,45]. The positive correlation between T_REG_ in treatment-naïve OCR and OFA patients at baseline and EDSS at m1 in our cohort could indicate that the OCR- or OFA-mediated increase in T_REG_s might be especially beneficial for patients with low T_REG_ counts at baseline.

DN T cells, which lack CD4 and CD8 molecules necessary for stabilizing T-cell receptor-major histocompatibility complex (TCR-MHC) interactions, are involved in non-classical antigen presentation primarily through CD1 molecules [43]. These cells exhibit natural suppressor activity that is not MHC-restricted, and their activation and regulatory functions are thought to be influenced by direct cell-to-cell contact and TCR signaling, rather than cytokine mediation [46]. DN T cells are highly activated in autoimmune diseases and have been implicated in conditions such as SLE, rheumatoid arthritis, type 1 diabetes, and Sjögren’s syndrome [44,45]. These cells are associated with increased inflammation and tissue damage, particularly in systemic lupus erythematosus (SLE) [47,48,49,50]. Using unsupervised clustering of the CD3^+^ T-cell population, we identified three distinct subsets of DN T cells, which were notably reduced in OCR- and OFA-treated RRMS patients compared to tnRRMS patients. Specifically, the percentage of KLRG1^−^HLADR^+^ DN T cells decreased significantly in OFA-treated patients at m12 compared to tnRRMS patients. Additionally, both KLRG1^+^HLADR^−^ DN T cells and KLRG1^+^HLADR^+^ DN T cells were significantly reduced in patients treated with B-cell depletion therapy at both m1 and m12, suggesting a sustained impact on these DN T cell subsets over time. Moreover, we found that a significantly higher proportion of DN T cells expressed CD20 compared to CD4^+^ and CD8^+^ T cells, providing a potential explanation for their reduction following OCR and OFA treatment. The reduction in DN T cells in our study aligns with an anti-inflammatory reconstitution of the immune system following B-cell depletion, with therapies possibly directly or indirectly reducing pathogenic DN T-cell populations in RRMS. Furthermore, the percentage of KLRG1^−^HLADR^+^ DN T cells was positively correlated with the number of relapses prior to baseline and negatively correlated with EDSS in ofatumumab-treated patients at m12, suggesting that this specific DN T-cell subset may be involved in disease activity and relapse frequency in MS. The reduction in this subset following B-cell depletion may therefore contribute to the overall clinical efficacy of OCR and OFA in reducing MS relapse rates.

## 5. Conclusions

Our 12-month interim analysis provides insight into immune reconstitution following B-cell depletion with OCR and OFA in MS. Apart from the expected decrease in CD20^+^ B and T cells, we observed an expansion of CD5^+^CD19^+^ B cells and an increase in exhaustion/activation markers like CTLA-4, PD-1, TIGIT, and TIM-3 across various immune cell types. Furthermore, OCR- and OFA-treatment led to an increase in T_REG_ and a reduction in DN T cells. The correlations we found between CD5^+^ B cells and clinical parameters, such as reduced disability scores and longer periods since the last relapse, suggest a potentially protective role for these cells in the disease course. The increased expression of exhaustion markers across various immune cells following B-cell depletion indicates a shift towards an immunological state characterized by reduced inflammatory potential. The significant reduction in DN T cells following treatment suggests that OCR and OFA may help diminish a potentially pathogenic cell population involved in MS progression. A better understanding of the specific functions and regulatory mechanisms of DN T cells and CD5^+^ B-cell subsets, as well as the implications of increased exhaustion marker expression, could optimize treatment strategies. Additionally, exploring how these therapies influence the balance between pro-inflammatory and regulatory roles in these cell populations could provide valuable insights toward improving the treatment of MS patients.

## 6. Limitations

Our study is limited by the small patient cohort. Due to the small sample size of patients with available multidimensional flow cytometry data from all three time points (m0, m1, and m12) no longitudinal analyses were performed. The aim of this current study was to provide an exploratory analysis of how the immune system reconstitutes following B-cell depletion, and future data will address in a larger and statistically higher-powered cohort why these changes occur. Moreover, the expression of CD5 and the exhaustion/activation markers CTLA-4, PD-1, TIM-3, and TIGIT on B cells is measured against the entire B-cell population, and not subpopulations of B cells due to the limited number of channels in conventional flow cytometry. Therefore, it is possible that the depletion of CD20^+^ B cells artificially increases other B-cell populations.

## Figures and Tables

**Table 1:** Basic demographic and disease characteristics of OCR, OFA, and treatment-naïve RRMS patients.

	OCR	OFA	tnRRMS
Total n.o. patients	34	25	20
N.o. patients at m1	16	22	NA
N.o. patients at m12	24	17	NA
Age (median [range])	36 [19–55]	37 [22–54]	37 [19–57]
Sex (% female)	71	80	90
Disease duration (median [range]) (Y)	3.42 [0.17–22.08]	3.42 [0.08–25.33]	0.63 [0.00–19.67]
EDSS at BL (median [range])	2.00 [0.0–6.5]	2.00 [0.0–3.5]	1.25 [0.0–5.0]
ARR at BL (median [range])	1 [0–2]	1 [0–3]	1 [0–3]
N.o. previous DMTs (median [range])	1 [0–4]	1 [0–5]	0 [0]

Basic demographic and disease characteristics of OCR, OFA, and treatment-naïve RRMS patients. Baseline characteristics of ocrelizumab (OCR)-receiving (n = 34), ofatumumab (OFA)-treated (n = 25), and treatment-naïve relapsing-remitting multiple sclerosis patients (tnRRMS) (n = 20). Median ages were 36, 37, and 37 years, respectively. Female patients comprised 71% (OCR), 80% (OFA), and 90% (tnRRMS) of each group. Median disease duration was 3.42 years for both OCR-treated and OFA-treated patients, and 0.63 years for tnRRMS. Median baseline EDSS scores were 2.00 (OCR), 2.00 (OFA), and 1.25 (tnRRMS). Median baseline annualized relapse rate (ARR) was 1 in all groups. Median prior disease-modifying therapies (DMTs) were 1 (OCR, OFA) and 0 (tnRRMS). Further details are in Supplementary Table S2. ARR—Annualized Relapse Rate; BL—Baseline; DMTs—Disease Modifying Therapies; EDSS—Expanded Disability Status Scale; N.o.—Number of; OCR—ocrelizumab; OFA—ofatumumab; tnRRMS—treatment-naïve relapsing remitting multiple sclerosis; Y—years.

## Data Availability

Data underlying this study are registered with the ABCD-J data catalog at https://data.abcd-j.de/dataset/b611539b-02f7-5008-bc21-3f3bb4a3dfcd/1.0, accessed on 27 March 2025. Further information, resources, anonymized clinical and flow cytometry data can be requested via the catalog item and will be fulfilled by Saskia Räuber (saskiajanina.raeuber@med.uni-duesseldorf.de).

## Supplementary Materials

The following supporting information can be downloaded at: https://www.mdpi.com/article/10.3390/cells14070552/s1, Figure S1: Exemplary gating of panel 1 B cell; Figure S2: Exemplary gating of panel 2 exhaustion; Figure S3: Exemplary gating of panel 3 T cell I; Figure S4: Exemplary gating of panel 4 ILC & NK; Figure S5: Exemplary gating of panel 5 T cell II; Table S1: Overview of the used mFC panels; Table S2: Additional clinical data of OCR, OFA, and treatment-naïve RRMS patients.

## Abbreviations

tnRRMS	treatment-naïve relapsing-remitting multiple sclerosis
OFA	ofatumumab
OCR	ocrelizumab
CSF	cerebrospinal fluid
m0	baseline, prior to treatment
m1	month 1 of treatment
m12	month 12 of treatment
TREG	T regulatory cell
NK cell	natural killer cell
NKT cell	natural killer T cell
ADCC	antibody-dependent cellular cytotoxicity
CDC	complement-dependent cytotoxicity
PBMC	peripheral blood mononuclear cell
mFC	multidimensional flow cytometry
RRMS	relapsing-remitting multiple sclerosis
IgG	immunoglobulin G
HIV	human immunodeficiency virus
