# Supplementary material for: Effects of Anti-CD20 Antibody Therapy on Immune Cell Dynamics in Relapsing-Remitting Multiple Sclerosis
Source: Cells. 2025 Apr 6;14(7):552. doi: 10.3390/cells14070552 (PMC11988809; doi:10.3390/cells14070552)
Supplement: Supplementary file 1 [file cells-14-00552-s001.zip › Supp Table S2.pdf]

Supplementary Table S2

Patient-ID	Group	Age	Sex	EDSS at baseline	Total numer of relapses	ARR at baselien	Time since disease manifestation (years)	Time since first diagnosis (years)	Time since last relapse (months)	Prev. number of DMTs	Last DMT	Reason for treatment change
OFA-1	OFA	39	F	1.0	14	2	6.50	6.1	1.7	1	GLAT	Escalation
OFA-2	OFA	23	F	1.0	3	1	3.00	3.0	3.4	1	GLAT	Escalation
OFA-3	OFA	36	F	2.0	5	1	7.25	6.3	5.9	5	CLAD	Escalation
OFA-4	OFA	38	F	1.0	3	2	1.33	1.0	1.8	1	DMF	Escalation
OFA-5	OFA	45	F	3.5	2	1	3.33	0.2	6.2	0	None	First DMT
OFA-6	OFA	27	F	1.5	2	1	3.42	1.6	1.0	0	None	First DMT
OFA-7	OFA	54	F	2.0	NA	1	25.33	22.8	10.7	1	IFN	Escalation
OFA-8	OFA	39	F	2.0	7	1	19.42	18.4	9.0	3	NAT	Escalation
OFA-9	OFA	43	M	1.0	3	2	0.08	0.0	1.3	1	TFL	Escalation
OFA-10	OFA	25	F	2.0	5	0	8.75	8.8	18.0	5	NAT	Positive JCV ab index
OFA-11	OFA	24	F	1.5	4	1	4.75	4.8	6.7	1	NAT	Escalation
OFA-12	OFA	32	F	2.0	2	1	8.58	8.6	11.1	4	FINGO	escalation
OFA-13	OFA	29	F	0.0	2	1	9.50	0.2	3.7	0	None	First DMT
OFA-14	OFA	51	F	2.0	4	1	6.75	4.7	4.2	2	FINGO	Escalation
OFA-15	OFA	24	M	2.0	4	1	5.50	4.5	2.8	1	GLAT	Escalation
OFA-16	OFA	53	F	2.0	2	1	1.25	0.2	2.7	3	TFL	Escalation
OFA-17	OFA	37	F	2.5	1	1	0.58	0.3	7.8	0	None	First DMT
OFA-18	OFA	39	F	2.5	1	1	0.33	0.3	4.2	0	None	First DMT
OFA-19	OFA	26	F	1.0	1	1	2.25	1.9	26.9	0	None	First DMT

Supplementary Table S2

OFA-20	OFA	26	F	2.0	1	1	0.33	0.3	3.9	0	None	First DMT
OFA-21	OFA	38	M	0.0	1	1	0.42	0.0	5.8	0	None	First DMT
OFA-22	OFA	37	F	2.0	2	1	12.00	0.1	3.1	0	None	First DMT
OFA-23	OFA	22	F	1.0	2	1	0.33	0.3	4.2	0	None	First DMT
OFA-24	OFA	NA	M	2.5	5	3	2.92	2.9	1.2	1	TFL	Escalation
OFA-25	OFA	47	M	1.0	2	0	12.17	5.2	9.8	2	GLAT	NA
OCR-1	OCR	23	F	3.5	2	2	0.58	0.5	3.1	0	None	First DMT
OCR-2	OCR	24	F	0.0	3	0	2.25	2.1	27.8	1	DMF	Positive JCV ab index
OCR-3	OCR	45	M	1.5	1	1	0.58	0.3	7.9	0	None	First DMT
OCR-4	OCR	36	F	1.0	3	1	6.33	0.2	4.3	0	None	First DMT
OCR-5	OCR	32	F	1.5	3	1	5.33	5.3	12.3	2	TFL	Escalation
OCR-6	OCR	41	F	2.0	4	1	21.42	21.4	3.7	3	DMF	Adverse effects
OCR-7	OCR	42	F	2.0	5	2	17.58	16.6	5.2	1	IFN	Escalation
OCR-8	OCR	28	F	2.0	1	1	0.67	0.7	8.9	0	None	First DMT
OCR-9	OCR	28	F	NA	1	1	1.17	1.2	14.1	0	None	First DMT
OCR-10	OCR	30	F	1.0	0	1	NA	0.8	NA	0	None	First DMT
OCR-11	OCR	38	F	3.5	6	1	14.75	10.0	2.5	2	IFN	Escalation
OCR-12	OCR	32	M	1.0	1	0	3.42	3.4	41.8	2	NAT	Positive JCV ab index
OCR-13	OCR	53	M	6.0	2	1	4.17	0.3	5.3	0	None	First DMT
OCR-14	OCR	52	F	3.0	2	2	0.17	0.1	2.7	0	None	First DMT

Supplementary Table S2

OCR-15	OCR	33	F	2.5	8	1	11.92	7.8	4.0	4	NAT	Escalation
OCR-16	OCR	45	F	3.5	3	0	8.83	8.8	52.4	1	IFN	Adverse effects
OCR-17	OCR	39	F	4.0	NA	0	20.83	20.8	46.8	2	NAT	Positive JCV ab index
OCR-18	OCR	34	F	2.0	6	2	3.33	3.3	3.2	1	TFL	Escalation
OCR-19	OCR	38	F	0.0	3	0	10.67	10.6	46.4	3	FINGO	Escalation
OCR-20	OCR	27	F	1.0	1	0	4.00	2.0	48.9	1	NAT	Escalation
OCR-21	OCR	31	F	3.0	2	1	1.17	0.5	2.8	1	GLAT	NA
OCR-22	OCR	44	M	2.0	2	2	0.50	0.4	4.8	1	NAT	NA
OCR-23	OCR	52	M	2.0	5	0	12.83	12.3	5.4	2	DMF	NA
OCR-24	OCR	26	F	2.5	2	1	0.17	0.2	2.3	0	None	0
OCR-25	OCR	30	M	1.0	1	1	0.42	0.4	5.1	0	None	0
OCR-27	OCR	40	M	6.5	5	1	11.50	11.5	7.0	4	GLAT	Escalation
OCR-28	OCR	22	M	1.0	2	1	2.50	0.2	2.4	0	None	First DMT
OCR-29	OCR	44	F	6.0	4	1	12.75	2.5	2.4	3	FINGO	Escalation
OCR-30	OCR	55	F	1.0	1	0	1.08	0.3	13.2	0	None	First DMT
OCR-31	OCR	19	F	1.0	1	1	0.00	12.5	0.8	0	None	NA
OCR-32	OCR	39	M	NA	2	1	2.50	0.3	4.1	0	None	First DMT
OCR-33	OCR	36	F	2.0	1	1	1.17	0.0	1.4	0	None	First DMT
OCR-34	OCR	43	F	5.5	7	1	22.08	12.1	5.6	2	FINGO	Escalation
OCR-35	OCR	31	M	2.0	7	2	5.08	5.1	1.5	2	TFL	Escalation
RRMS-1	RRMS	39	M	5.0	2	1	1.58	1.6	0.0	0	None	NA

Supplementary Table S2

RRMS-2	RRMS	39	F	2.0	3	2	0.58	0.6	4.9	0	None	NA
RRMS-3	RRMS	57	F	2.0	NA	NA	18.67	18.7	0.0	0	None	NA
RRMS-4	RRMS	34	F	2.0	2	1	1.33	1.3	NA	0	None	NA
RRMS-5	RRMS	38	F	1.5	1	1	0.67	0.7	8.2	0	None	NA
RRMS-6	RRMS	36	F	NA	3	2	0.00	0.0	0.1	0	None	NA
RRMS-7	RRMS	33	F	1.0	2	1	0.00	0.0	0.2	0	None	NA
RRMS-8	RRMS	24	M	2.0	NA	NA	0.17	0.2	0.4	0	None	NA
RRMS-9	RRMS	35	F	2.0	1	0	0.08	0.1	1.3	0	None	NA
RRMS-10	RRMS	20	F	1.0	NA	NA	2.08	2.1	0.0	0	None	NA
RRMS-11	RRMS	39	F	1.0	4	0	19.67	19.7	25.5	0	None	NA
RRMS-12	RRMS	19	F	1.0	1	1	0.42	0.4	5.3	0	None	NA
RRMS-13	RRMS	22	F	1.0	1	0	0.25	0.3	2.8	0	None	NA
RRMS-14	RRMS	45	F	NA	NA	NA	0.17	0.2	0.0	0	None	NA
RRMS-16	RRMS	29	F	0.0	2	2	0.75	0.8	7.5	0	None	NA
RRMS-15	RRMS	38	F	2.5	0	0	4.50	4.5	NA	0	None	NA
RRMS-17	RRMS	44	F	0.0	1	0	2.17	2.2	2.4	0	None	NA

Supplementary Table S2

RRMS-18	RRMS	30	F	2.0	1	0	0.00	0.0	0.0	0	None	NA
RRMS-19	RRMS	44	F	1.0	2	2	2.00	2.0	2.8	0	None	NA
RRMS-20	RRMS	46	F	1.0	1	0	0.33	0.3	0.0	0	None	NA
