# Supplementary figures and images for: Effects of Anti-CD20 Antibody Therapy on Immune Cell Dynamics in Relapsing-Remitting Multiple Sclerosis
Source: Cells. 2025 Apr 6;14(7):552. doi: 10.3390/cells14070552 (PMC11988809; doi:10.3390/cells14070552)

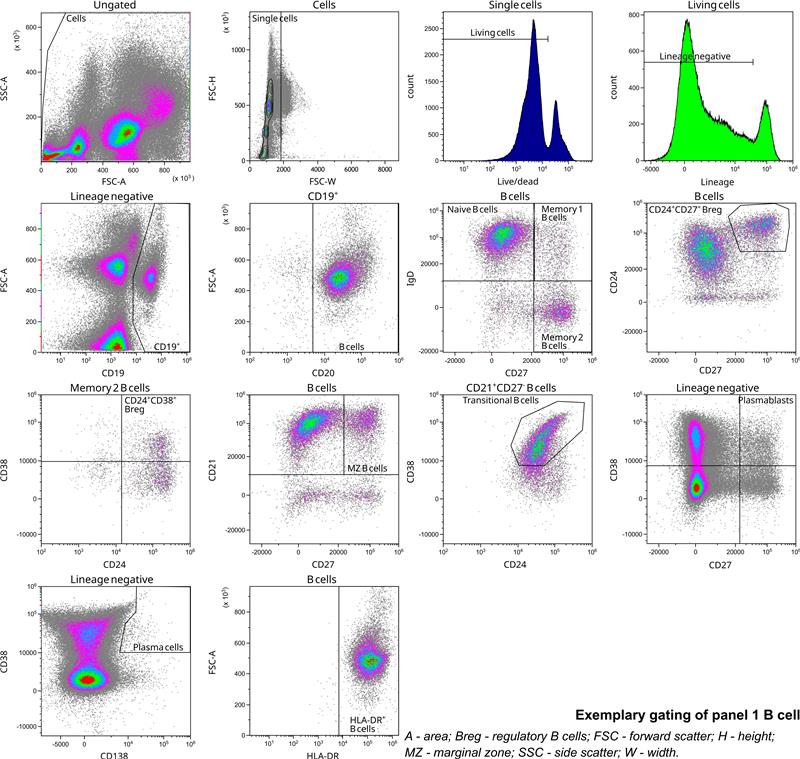

Supplement: Supplementary file 1 [file cells-14-00552-s001.zip › Supp Figure S1.png]

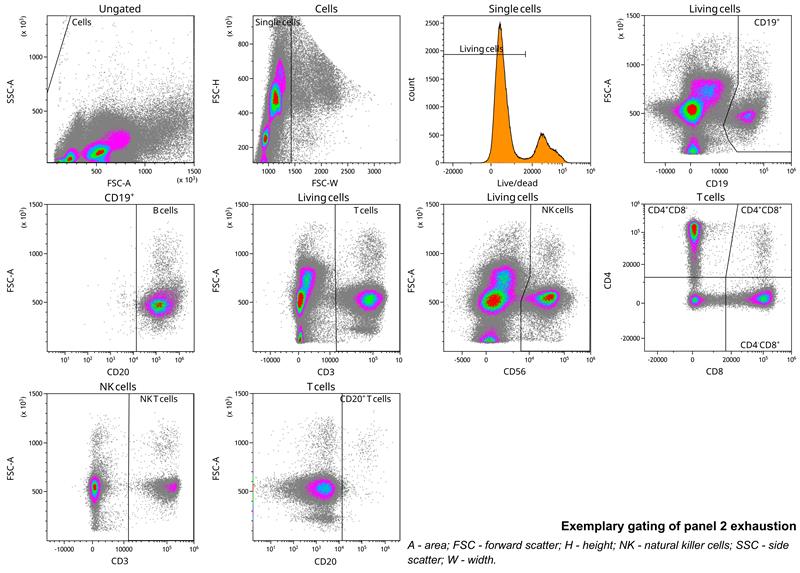

Supplement: Supplementary file 1 [file cells-14-00552-s001.zip › Supp Figure S2.png]

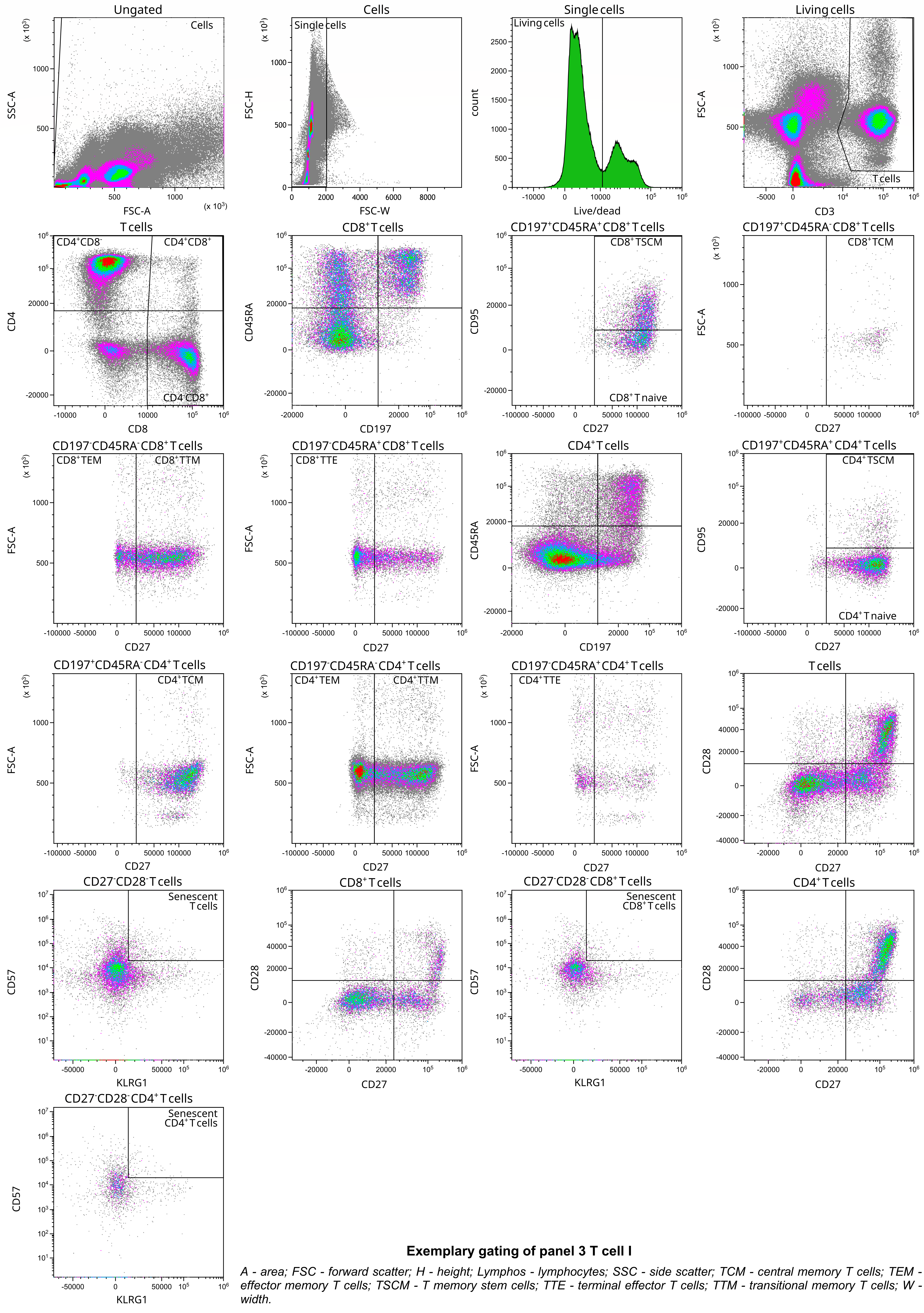

Supplement: Supplementary file 1 [file cells-14-00552-s001.zip › Supp Figure S3.png]

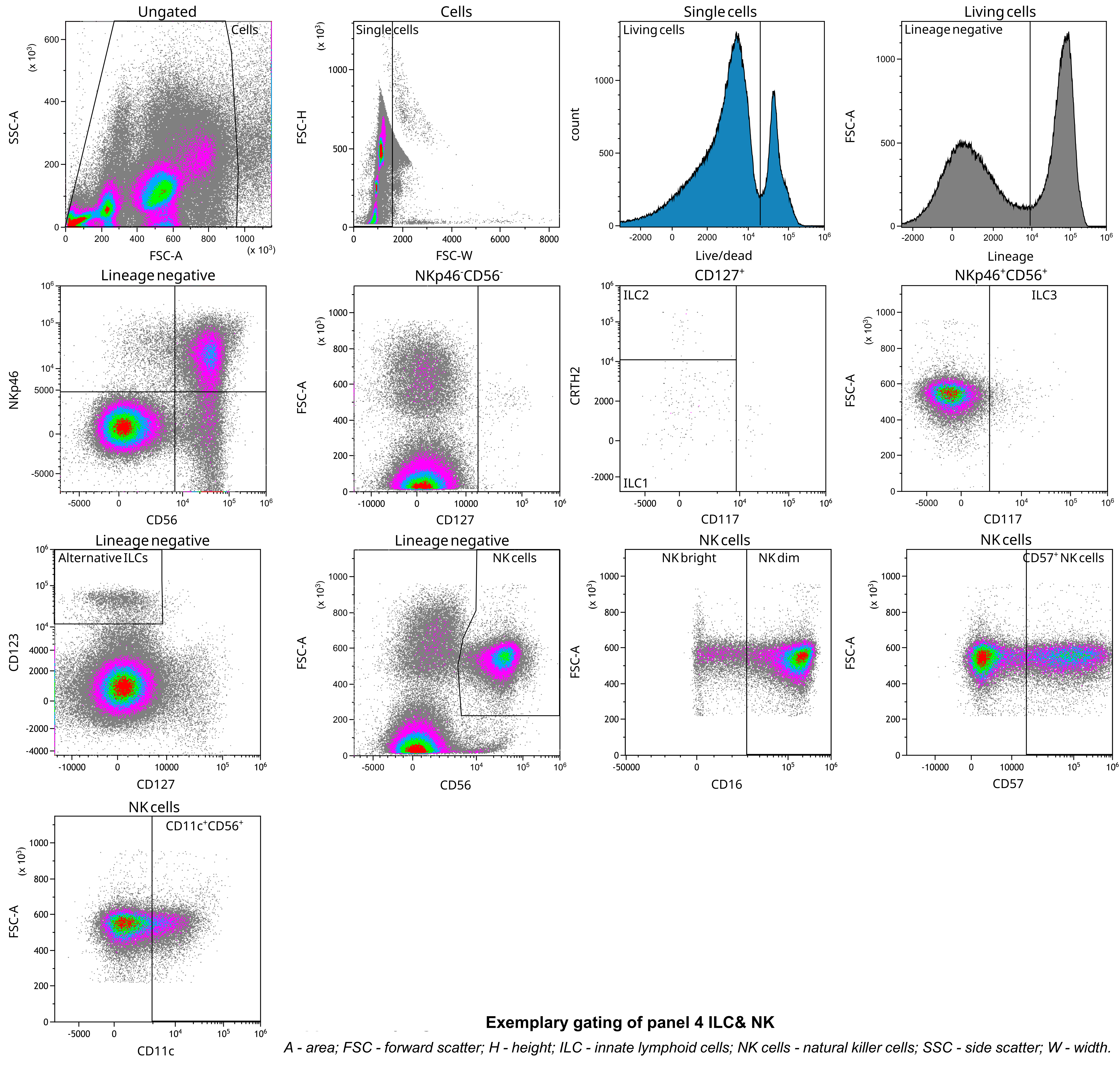

Supplement: Supplementary file 1 [file cells-14-00552-s001.zip › Supp Figure S4.png]

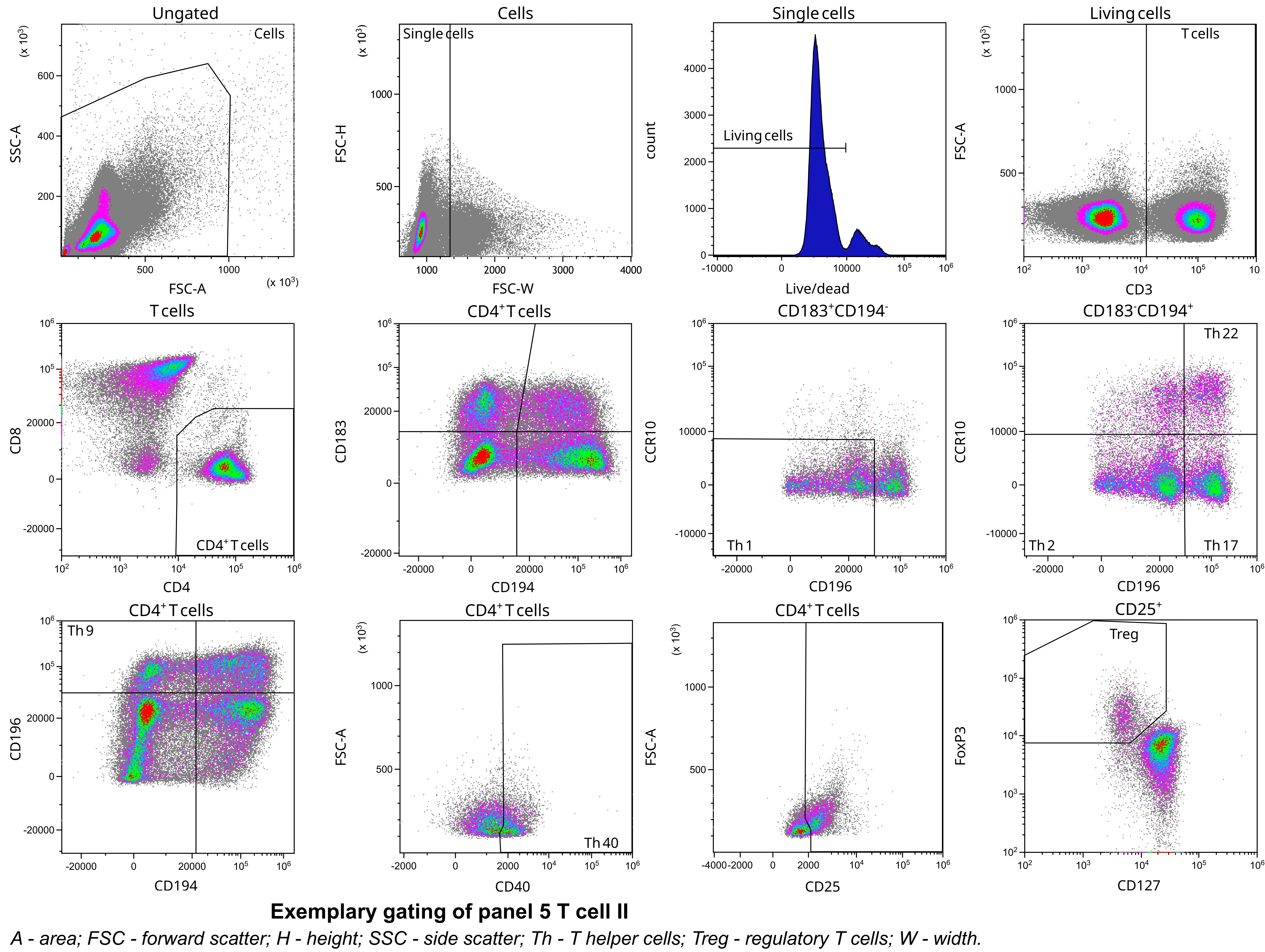

Supplement: Supplementary file 1 [file cells-14-00552-s001.zip › Supp Figure S5.png]
